# A Further Study of Perforation of the Bowel in Typhoid Fever

**Published:** 1907-03

**Authors:** 


### A FURTHER STUDY OF PERFORATION OF THE BOWEL IN TYPHOID FEVER.

Continuing his study of this subject, J. A. Scott, M. D., presents the following conclusions:

Perforation of the bowel in typhoid fever is more common than is generally supposed, occurring once and a trifle over in every three deaths.

The most common time of perforation is between the fourteenth and twenty-first days. In 92 per cent. of the cases in this series the perforation occurred between the second and fifth week inclusive. The earlier cases are probably perforation in a relapse; now and then perforation may occur without evidence of previous illness.

Perforation occurs in cases of all grades of severity, from the ambulatory to the hæmorrhagic type. It is most common in those with moderate (25 per cent.) and severe (50 per cent.) infection (75 per cent.). It is more common in the hæmorrhagic than in the mild cases (10.8 per cent. to 8 per cent.).

The ileum is the common site of perforation (88 per cent.; the majority occur within twelve inches of the ileocæcal valve; the appendix and colon, respectively, are the next most frequent sites of perforation in this series of cases.

Pain of some kind is present in 75 per cent. of all cases. In 50 per cent. of the cases the onset is sudden and severe and of increasing intensity, localizing itself to a special zone. In 20 per cent. of the cases the pain is of slow onset, not localized, with general distribution. In some cases (12 per cent. of this series) no pain is complained of, and the usual symptoms of perforation are absent.

Tenderness and rigidity are present in from 75 to 65 per cent., respectively, of all cases, and are usually combined; in some cases either one or the other may be wanting; rigidity especially may be absent in cases with rather a pendulous and relaxed abdominal wall.

When perforation is suspected the temperature should be taken every hour; only by this means can the immediate rise and slow fall to normal or subnormal which often occurs be detected; in some cases, and especially those of extreme toxicity, no noteworthy change at all in the pulse, temperature, or respiration can be detected when perforation occurs. Diagnosis is then only an inference.

Distention (if absent during the course of the disease and at the time of suspected perforation) is a late symptom of perforation. The obliteration of liver dulness is not a reliable sign of perforation.

The study of the leucocytes is of little aid. In a few cases their increase is such as to assure you of your diagnosis. In a considerable number of cases there is a decided reduction in leucocytes after symptoms of perforation. Differential counting is not of practical use.

Before being assured of our diagnosis right sided pleurisy, pneumonia (especially in the young), cholecystitis, acute gastrointestinal indigestion, femoral and iliac thrombosis, appendicitis, peritonitis without perforation, cystitis, rupture of a mesenteric gland, or even hæmorrhagic exudation into the abdominal muscles (Zenker's degeneration) should be considered. Even then mistakes in diagnosis will be made.

While nature will infrequently close one, two, or even three perforations, the only rational procedure when perforation occurs is operative interference. No case is too desperate for the attempt. Not infrequently the so-called mild cases succumb, while very ill ones recover.

The diagnosis made, time for operation has arrived; its important point is rapidity. Closure of the perforation and drainage is all that is needed; fifteen to twenty minutes should suffice.—New York Medical Journay, February 9th, 1907.

